# Supplementary material for: Altered coagulation and platelet indices in Yemeni patients with type 2 diabetes mellitus: A conflict-affected population
Source: PLOS Glob Public Health. 2026 Jan 21;6(1):e0005173. doi: 10.1371/journal.pgph.0005173 (PMC12822953; doi:10.1371/journal.pgph.0005173)
Supplement: S1 File — Structured questionnaire used to collect demographic and clinical data. (DOCX) [file pgph.0005173.s001.docx]

Republic of Yemen

University of Sciences and Technology Faculty of Medicine and Health Sciences Department of Health Sciences

S1 File: Data Collection Questionnaire Form (English Version)

Protocol Title:

The Paradox of War and Diabetes: Prolonged Prothrombin Time, Shortened APTT, and Platelet Activation in Yemeni Patients with Type 2 Diabetes Mellitus

Data Collection Questionnaire (English Translation)

Study ID: _________

SECTION A: DEMOGRAPHICS

1. Date of Interview: ____/____/2025

2. Age (Years): ______

3. Sex: [ ] Male [ ] Female

4. Contact Phone Number (Optional): ________________

SECTION B: MEDICAL HISTORY (To be confirmed with patient file)

5. Diagnosis of Type 2 Diabetes Mellitus: [ ] Yes [ ] No (If No, stop here for controls)

6. Duration of Diabetes (Years): ______

7. Current Diabetic Medications:

- [ ] Metformin

- [ ] Sulfonylurea (e.g., Glibenclamide)

- [ ] Insulin

- [ ] Other (Specify): _________________

8. History of Diabetic Complications:

- Retinopathy (Eye disease): [ ] Yes [ ] No [ ] Unknown

- Nephropathy (Kidney disease): [ ] Yes [ ] No [ ] Unknown

- Neuropathy (Nerve disease): [ ] Yes [ ] No [ ] Unknown

9. History of Thrombotic Events (Heart attack, stroke, DVT): [ ] Yes [ ] No [ ] Unknown

10. Current Use of Antiplatelet/Anticoagulant drugs (e.g., Aspirin, Clopidogrel, Warfarin): [ ] Yes [ ] No

11. Any other major chronic illness? [ ] Yes (Specify: _______________) [ ] No

SECTION C: FOR ALL PARTICIPANTS

12. Fasting Time Confirmed (>12 hours): [ ] Yes [ ] No

13. Blood Sample Drawn: [ ] Yes [ ] No

14. Sample Time: ________ (24h time)

Investigator's Name & Signature: _________________________
