# Supplementary material for: Altered coagulation and platelet indices in Yemeni patients with type 2 diabetes mellitus: A conflict-affected population
Source: PLOS Glob Public Health. 2026 Jan 21;6(1):e0005173. doi: 10.1371/journal.pgph.0005173 (PMC12822953; doi:10.1371/journal.pgph.0005173)
Supplement: S2 File — Detailed description of the logistical and infrastructural challenges faced during the study, including electricity supply issues, reagent scarcity, and healthcare access limitations. (DOCX) [file pgph.0005173.s002.docx]

Republic of Yemen

University of Sciences and Technology Faculty of Medicine and Health Sciences Department of Health Sciences

S2 File: Contextual Challenges Appendix

The Paradox of War and Diabetes: Prolonged Prothrombin Time, Shortened APTT, and Platelet Activation in Yemeni Patients with Type 2 Diabetes Mellitus

Contextual Challenges Appendix

Detailed Description of Contextual Challenges

The data for this study was collected amidst the severe and ongoing healthcare collapse in Yemen, which actively shaped the research process and findings.

1. Infrastructure: Intermittent electricity supply was a constant challenge. Blood samples were processed using backup generators to ensure the 2-hour processing window was met, preventing sample degradation.

2. Reagent Scarcity: Laboratory reagents for the STA-R Evolution and Sysmex analyzers were often scarce, requiring careful rationing and prioritization of tests. This occasionally delayed non-essential analyses.

3. Patient Access to Care: As noted in the cohort, consistent diabetic management was a rarity. Access to basic medications like insulin was often interrupted, and advanced anticoagulant therapy was virtually non-existent. Less than 15% of our diabetic cohort was on regular aspirin, a stark indicator of the therapeutic gap.

4. Nutritional Context: Widespread food insecurity and malnutrition are well-documented in this population. This contextual factor is not merely a background note but is hypothesized to be a direct contributor to the paradoxical coagulation profile (prolonged PT) observed, likely through deficiency of fat-soluble vitamins like Vitamin K.

These challenges underscore that the observed hemostatic profile is a result of the disease (T2DM) interacting with a unique and extreme environment, making these findings critically important for humanitarian medicine.
