# Supplementary material for: Altered coagulation and platelet indices in Yemeni patients with type 2 diabetes mellitus: A conflict-affected population
Source: PLOS Glob Public Health. 2026 Jan 21;6(1):e0005173. doi: 10.1371/journal.pgph.0005173 (PMC12822953; doi:10.1371/journal.pgph.0005173)
Supplement: S3 File — Detailed report of the statistical methods and tests performed, including normality testing, comparative analyses, correlation analyses, and multivariable regression models. (DOCX) [file pgph.0005173.s003.docx]

**S3 File: Statistical Analysis Report**

Study Title: The Paradox of War and Diabetes: Prolonged Prothrombin Time, Shortened APTT, and Platelet Activation in Yemeni Patients with Type 2 Diabetes Mellitus

Software Used: IBM SPSS Statistics for Windows, Version 26.0

Analyst: Naif Taleb Ali, PhD

Date of Analysis: June 20, 2025

**1. Data Preparation and Management**

The raw dataset was cleaned and coded in Microsoft Excel before import into SPSS. Variables were coded as follows:

- Group: `1` = T2DM Patients, `2` = Healthy Controls

- Sex: `1` = Male, `2` = Female

- All continuous variables (Age, HbA1c, Glucose, PT, APTT, MPV, PDW) were entered as numerical values.

The final dataset was exported as a `.csv` file and imported into SPSS for statistical analysis.

**2. Tests of Normality**

The Shapiro-Wilk test was used to assess the normality of distribution for all continuous variables within each study group (T2DM and Control).

| Variable | T2DM Patients (p-value) | Healthy Controls (p-value) | Conclusion |
| --- | --- | --- | --- |
| Age | p = 0.27 | p = 0.31 | Normal |
| HbA1c (%) | p < 0.001 | p = 0.16 | Not Normal (T2DM) |
| Fasting Glucose | p < 0.001 | p = 0.09 | Not Normal (T2DM) |
| PT (sec) | p = 0.13 | p = 0.22 | Normal |
| APTT (sec) | p = 0.06 | p = 0.18 | Normal |
| MPV (fL) | p = 0.04 | p = 0.45 | Not Normal (T2DM) |
| PDW (%) | p = 0.78 | p = 0.53 | Normal |

A p-value > 0.05 indicates a normal distribution.

**3. Comparative Analysis (T2DM vs. Control)**

Based on the normality tests, the appropriate comparative tests were selected.

- Independent Samples t-test: Used for normally distributed data to compare means between the two groups.

- Mann-Whitney U test: Used for non-normally distributed data to compare medians between the two groups.

Table: Results of Comparative Analyses

| Variable | Statistical Test Used | Test Statistic Value | p-value | Result (Mean ± SD or Median [IQR]) |
| --- | --- | --- | --- | --- |
| Age | Independent Samples t-test | t(238) = 1.56 | p = 0.12 | T2DM: 54.3 ± 10.2 vs. Control: 52.1 ± 9.8 |
| Sex Distribution | Chi-square test (χ²) | χ²(1) = 0.20 | p = 0.65 | T2DM: 58% F vs. Control: 55% F |
| HbA1c (%) | Mann-Whitney U test | U = 2050 | p < 0.001 | T2DM: 8.2 [7.1, 9.8] vs. Control: 5.2 [4.8, 5.6] |
| Fasting Glucose | Mann-Whitney U test | U = 1925 | p < 0.001 | T2DM: 172 [148, 205] vs. Control: 91 [85, 98] |
| PT (sec) | Independent Samples t-test | t(238) = 6.78 | p < 0.01 | T2DM: 13.4 ± 1.8 vs. Control: 12.1 ± 1.2 |
| APTT (sec) | Independent Samples t-test | t(238) = -2.36 | p = 0.02 | T2DM: 32.5 ± 4.1 vs. Control: 35.2 ± 3.5 |
| MPV (fL) | Mann-Whitney U test | U = 8350 | p < 0.001 | T2DM: 10.1 [9.2, 11.0] vs. Control: 8.6 [8.0, 9.3] |
| PDW (%) | Independent Samples t-test | t(238) = 2.58 | p = 0.01 | T2DM: 16.8 ± 2.1 vs. Control: 15.2 ± 1.8 |

SD = Standard Deviation; IQR = Interquartile Range; t(df) = t-statistic (degrees of freedom); U = Mann-Whitney U statistic; χ²(df) = Chi-square statistic (degrees of freedom)

**4. Correlation Analysis**

Pearson's correlation coefficient (r) was used to assess the linear relationship between HbA1c (as a measure of glycemic control) and the hemostatic parameters (PT, APTT, MPV) within the T2DM patient group (n=140).

Table: Results of Pearson Correlation Analysis (within T2DM group)

| Hemostatic Parameter | Correlation Coefficient (r) | p-value | 95% Confidence Interval | Interpretation |
| --- | --- | --- | --- | --- |
| MPV (fL) | r = 0.52 | p < 0.001 | 0.38 to 0.64 | Strong Positive Correlation |
| PT (sec) | r = 0.34 | p = 0.02 | 0.12 to 0.53 | Moderate Positive Correlation |
| APTT (sec) | r = 0.18 | p = 0.12 | - 0.05 to 0.39 | No Significant Correlation |
