# Supplementary material for: Altered coagulation and platelet indices in Yemeni patients with type 2 diabetes mellitus: A conflict-affected population
Source: PLOS Glob Public Health. 2026 Jan 21;6(1):e0005173. doi: 10.1371/journal.pgph.0005173 (PMC12822953; doi:10.1371/journal.pgph.0005173)
Supplement: S1 Checklist — This checklist is adapted from the STROBE Statement and is licensed under a Creative Commons Attribution 4.0 International license (https://creativecommons.org/licenses/by/4.0/). Original source: https://www.strobe-statement.org/. (DOCX) [file pgph.0005173.s004.docx]

# STROBE Statement—Checklist of items that should be included in reports of case-control studies

| Section/Topic | Item # | Recommendation | Page Number |
| --- | --- | --- | --- |
| Title and abstract | 1 | (a) Indicate the study's design with a commonly used term in the title or the abstract | 1 |
|  |  | (b) Provide in the abstract an informative and balanced summary of what was done and what was found | 1 |
| Introduction | 2 | Explain the scientific background and rationale for the investigation being reported | 2-3 |
| Background/rationale | 3 | State specific objectives, including any prespecified hypotheses | 3 |
| Methods | 4 | Present key elements of study design early in the paper | 4 |
| Study design | 5 | Describe the setting, locations, and relevant dates, including periods of recruitment, exposure, follow-up, and data collection | 4 |
| Setting | 6 | (a) Give the eligibility criteria, and the sources and methods of case ascertainment and control selection | 4-5 |
| Participants |  | (b) For matched studies, give matching criteria and the number of controls per case | 5 |
| Variables | 7 | Clearly define all outcomes, exposures, predictors, potential confounders, and effect modifiers. Give diagnostic criteria, if applicable | 4-5 |
| Data sources/ measurement | 8 | For each variable of interest, give sources of data and details of methods of assessment (measurement) | 5-6 |
| Bias | 9 | Describe any efforts to address potential sources of bias | 5 |
| Study size | 10 | Explain how the study size was arrived at | 5 |
| Quantitative variables | 11 | Explain how quantitative variables were handled in the analyses. If applicable, describe which groupings were chosen and why | 6 |
| Statistical methods | 12 | (a) Describe all statistical methods, including those used to control for confounding | 6 |
|  |  | (b) Describe any methods used to examine subgroups and interactions | 6 |
|  |  | (c) Explain how missing data were addressed | 6 |
|  |  | (d) If applicable, explain how loss to follow-up was addressed | - |
|  |  | (e) Describe any sensitivity analyses | - |
| Results | 13 | (a) Report numbers of individuals at each stage of study | 5 |
| Participants |  | (b) Give reasons for non-participation at each stage | 5 |
|  |  | (c) Consider use of a flow diagram | - |
| Descriptive data | 14 | (a) Give characteristics of study participants and information on exposures and potential confounders | 5, Table 1-2 |
|  |  | (b) Indicate number of participants with missing data for each variable of interest | 5 |
| Outcome data | 15 | Report numbers in each exposure category, or summary measures of exposure | 5-8 |
| Main results | 16 | (a) Give unadjusted estimates and confounder-adjusted estimates with precision | 7, Table 4 |
|  |  | (b) Report category boundaries when continuous variables were categorized | 7 |
|  |  | (c) If relevant, consider translating estimates of relative risk into absolute risk | - |
| Other analyses | 17 | Report other analyses such as subgroup analyses and sensitivity analyses | 6-8 |
| Discussion | 18 | Summarize key results with reference to study objectives | 9-11 |
| Key results | 19 | Discuss limitations of the study, considering bias and imprecision | 12 |
| Limitations | 20 | Give cautious overall interpretation considering objectives and evidence | 11-12 |
| Interpretation | 21 | Discuss the generalizability of the study results | 12 |
| Generalizability | 22 | Give the source of funding and role of funders | 15 |
