# Supplementary material for: Altered coagulation and platelet indices in Yemeni patients with type 2 diabetes mellitus: A conflict-affected population
Source: PLOS Glob Public Health. 2026 Jan 21;6(1):e0005173. doi: 10.1371/journal.pgph.0005173 (PMC12822953; doi:10.1371/journal.pgph.0005173)
Supplement: Attachment — Submitted filename: PGPH-D-25-02423_review Report.pdf [file pgph.0005173.s005.pdf]

Article title:

The Paradox of War and Diabetes: Prolonged Prothrombin Time, Shortened APTT, and Platelet Activation in Yemeni Patients with Type 2 Diabetes Mellitus.

Alternate title:

Coagulation Paradox in Yemeni T2DM Patients

Review Summary

The manuscript is generally well articulated and structured as expected of observational studies in clinical epidemiology. The authors exhibited good knowledge of the subject matter, and the findings has potential to contribute new understanding of the hypercoagulation profile of type-2 diabetic patients in the study setting, which may better inform the global efforts to control this increasingly challenging metabolic disease in similar context. Contrary to the sensational title that highlights war and the authors' narrative fixated on potential effects of war-induced malnutrition and disruptive access to essential healthcare services in a typical humanitarian setting, this manuscript did not report any assessment of variables that measures nutritional status, use or access to needed medications or treatment in the diabetic and non-diabetic Yemeni participants studied. Instead of speculative narration of what could have been, the authors had the luxury of measuring these important confounding nutritional and healthcare access factors of interest, adjust their analysis for them, in order to correctly make any valid allusions to their possible effects on the observed prolongation of PT in this study.

Unfortunately, nutritional status was not measured in this study, use of metformin that could alter bioavailability of vitamin k needed for endogenous synthesis of essential coagulation factors was also not measured. So, it is spurious to insinuate that every person in Yemen (by extension, the 240 persons studied at Aden) is suffering from inherent malnutrition by default because Yemen is a known conflict setting. No evidence in this manuscript shows malnutrition in the participants, especially undernutrition, and no variables was included to show whether they were using metformin or not. Hence, it is concerning that the un-adjusted atypical finding of prolonged average PT in the type-2 diabetic participants is being speculatively explained by the authors as some novel paradox due to war induced-malnutrition, when a simple stratified sub-analysis or multiple regression could reject or confirm that reasoning. It seems illogical to draw attention to very important confounding factors that could have been easily measured but were not measured, when such should be some anecdotal notes in the sub-section for study setting or discussion of study limitations.

However, this study clearly assessed some selected coagulation or haemostatic markers in a group of 140 hospital-based type-2 diabetes mellitus (T2DM) patients in Yemen, attempted to determine the crude effect of their glycaemic control status on these markers, and compared these findings with 100 age-sex-matched non-diabetic participants. The authors were not sure if they conducted a cross-sectional or a case-control study as they reported both as the study design. This is an unacceptable methodological anomaly which may be reflective of the inefficient use of more appropriate statistical techniques in their analysis, poor interpretation of their findings, and the inconsistent use of applicable technical terminologies (e.g. cases, controls, cohort, group, patients, participants, etc) in their narrative across the manuscript.

Based on the highlighted technical concerns, it is apparent that the study design, analysis and narrative will benefit from substantial modification. Accordingly, I am recommending a **major revision** of this manuscript before its acceptance for publication.

Details of the Issues in the Manuscript

To support the authors' revision efforts, it is hereby suggested that the authors should revise the manuscript to address the following highlighted concerns:

1) *Title & Abstract*

As currently written, the obvious paradox in this manuscript is the author's decision to leave out the measurement and adjustment for clearly important confounding factors of interest, which could have explained the observed prolongation of average PT among the diabetic patients. If the authors' wish to make this manuscript about war-induced crippling of the healthcare system and widespread malnutrition, it is basic that they measure variables that assesses these factors and adjust their analysis for them. Another paradox is the anomaly of defining the study design as cross-sectional in the abstract but, it is stated as cross-sectional and case-control in the main text. Also, who were the diabetic participants compared with to arrive at the claim of significance in the observed poor glycaemic control (86%) as reported under the result sub-section of the abstract? The adjectival term "significant" is inappropriate to express a descriptive summary of a variable. In its statistical or clinical meaning, a comparison is required to assert a significant difference. Lastly, the conclusive statement is more aspirational than anything that can be specifically deduced from the index study. In this study, participants who were currently using anti-coagulants or anti-platelets medications were excluded. Therefore, since the observed average PT of the diabetic participants were already prolonged in this study, why will this finding support any advocacy for more anti-thrombotic therapy in conflict settings when such will likely worsen the already prolonged PT? Accordingly, the title and abstract should be re-written to reflect what the real study objective, methods and findings were.

2) *Introduction and Study Objectives*

The authors argued in the second paragraph of the introduction that a prolonged PT is one of the typical markers seen among diabetic patients. First, this is not truly affirmed by the literature referenced. Secondly, if that were to be taken as is, the prolonged PT seen in the index study result would not be a paradox or abnormal finding to highlight. More correctly, hypercoagulability is NOT an inevitable marker of diabetes in all patients living with the disease. Rather, it is a common marker of "uncontrolled diabetes" that is directly explained by poor glycaemic control, where a shortened PT is the commonest finding but, a prolonged PT has been reported, as well. Again, these further questions the proclamation of some new paradox associated with war by this manuscript. The authors should revamp their argument to be more coherent and consistent with existing literature and their research questions.

In the 3rd paragraph, the authors highlighted that Yemen is experiencing a rising prevalence

of T2DM. This is consistent with global trend in virtually every country, with or without conflict or any disruption of access to healthcare services. Why are the authors still pushing the narrative of a possible conflict effect or explanation for a demographic transition and lifestyle change issue? More so, the participants in this study were recruited at tertiary-care diabetic clinic, implying that they had access to specialist diabetic care. A population-based study that included participants with no access to care or diagnosis might justify the generalization of no access to healthcare. Variables measuring degree of access to appropriate care and treatment must be measured and adjusted for to show if conflict-induced disruption of healthcare had any meaningful effect on the coagulation profile of the hospital-based participants studied.

In the 4th an 5th paragraph, the study aims were unnecessarily repeated without any added information. More importantly, this study did not report what can be defined as comprehensive coagulation parameters and platelet indices. The reported assessment of PT, APTT, MPV and PDW can best be labelled a selection of key haemostatic or coagulation indicators. Also, it may be more correct to state that this study simply assessed the crude or unadjusted association of selected coagulation parameters with glycaemic control status among type-2 diabetic and non-diabetic adults in Aden, Yemen. The authors should consider modifying the introduction and study objectives to highlight more relevant information with improved specificity to what was actually measured and studied.

3) Methods

A study design is literally a technical box that guides what can be done, how it is done and what can be inferred from the findings of a given research endeavour. Therefore, the authors cannot be ambivalent about what study design was used in this study. A cross-sectional study, even when analytic, is very different from a case-control study. It is comical for the authors to claim they did a cross-sectional, case-control study. This is a fundamental assertion that ridicules the research methodological tenacity of this manuscript. The authors need to state the specific epidemiological study design that was applied in the conduction of this study, which is the fulcrum around which every other aspect of their methodological approach and interpretation of the findings must revolve.

What is stated as contextual challenges under sub-section 2.2 will be best left as discursive points when highlighting limitations of methods and findings in the appropriate paragraphs under the discussion section. In any case, the authors need to be reminded that these general challenges cannot be summarily adopted to have affected this study if they cannot highlight exactly how each of the challenge limited the accuracy and precision of their methodological processes including the recruitment of study participants, assessment of variables of interest and the analysis of results. For example, how did the use of back-up generator change anything about this study? They said laboratory reagents were scarce but, if they got the right reagents and used them correctly, how did reagent scarcity affect the findings of this study? Also, the authors stated that *"patient access to consistent diabetic care and anticoagulant therapy was limited, as reported in our cohort, where less than 15% were on regular aspirin"*. I failed to find any variable in any aspect of this manuscript that reported this. So, what cohort are they referring to? Moreso, how is this a challenge to this study when in the following sub-section,

one of the exclusion criteria was *"use of anticoagulant or antiplatelet medication"*? Lastly, the authors boldly declared that: *"These contextual factors are not just background, but active determinants of the hemostatic profile observed, reflecting a reality where disease pathophysiology is inextricably linked to systemic healthcare failure"*. For the umpteenth time, as I have highlighted repeatedly, if these factors are perceived to be important determinant of haemostatic profile, then they should have been defined as confounding variables, measured, included in the analysis and adjusted for. However, some of the information about contextual challenges can be better re-phrased under study setting.

Concerning sub-section 2.3, sample size estimation formulars are study design specific. With the authors' ambivalence about the specific epidemiologic study design applied, the correctness of the quoted minimum sample size is questionable. Howbeit, no reference was provided for the goal to detect a clinically significant difference in MPV of 1.0fl. Is this an arbitrary choice? Ideally, a minimum sample size should be estimated for each main independent variable of interest, and the largest minimum will be adopted as the rational choice as the minimum sample size, to ensure the studied number of participants are adequate to detect the anticipated difference for all key variables. MPV is only one of many other factors of interest in this study, is there any reason for the choice of MPV as the sample size estimation variable? More concerning is the fact that MPV is one of the outcome variables in this study, as portrayed. The variable(s) to be used to estimate sample size should the predictor (independent) variables that you wish to estimate their effect(s) on the outcome. Also, the authors will need to clearly specify and define the outcome (dependent) and predictor (independent) variables for this study under an appropriate sub-heading. Assuming the estimated sample size is correct, 120 participants were needed in each group. Statistically, increasing the number of non-diabetic (healthy controls) is justifiable but, ethically, it is unusual to increasing the number of diabetic (ill persons) while reducing the number of controls. The authors quickly claimed *"well-documented constraints of the research setting"* for this but, they need to be more specific about what constraint made it easier to find more type 2 diabetic patients than healthy controls for this study in Aden.

About the study participants, the 140 type-2 diabetic patients were reportedly enlisted from an outpatient clinic but, the use of a simple random sampling technique in require a few more details. To claim simple random sampling was truly done, the authors are affirming that they had a sampling frame and line-list of the total patients. So, they should report the total population size of the type-2 DM patients attending that clinic, and how they randomly selected 140. The non-diabetic participants, whom the authors tagged "??healthy controls", seemed to have qualified as "healthy" by their personal medical history profile alone. Are the authors saying they relied on the validity of self-reported medical history to ascertain if they were healthy, free of type-2 diabetes and other diseases? The least requirement should be some clinical assessment by a licensed physician if other screening investigations are two expensive for the research budget. Also, the post-hoc fasting glucose levels shown in their results table-1 could suffice as proof that they were not diabetic. More importantly, the authors were silent about the source of these "healthy controls". Were they other patients attending the same hospital for other reasons or some wider community members? How were they selected? Did they apply simple random sampling technique, too? Knowing the study-base for the control

group is a very important methodological information. The general inclusion and exclusion criteria that applied to the diabetic group should be applied to the non-diabetic (healthy) group, except that the former should be diagnosed of type-2 diabetes.

I will suggest that sub-section 2.5 should be expanded to define the key variables measured in this study, so that it can be abundantly clear whether your use of those terms is the same or different from the literature definitions. It is also the right place to highlight the outcome, main predictor(s) and control variables. Information about blood sample collection can be moved under laboratory procedure or technique (subsection2.6). Finally, the statistical analysis narrative is generic and will certainly need to be re-written, after the authors have chosen a specific study design and defined their variables clearly. Irrespective of whether they did a cross-sectional or a case-control study, the effect of glycaemic control on the observed haemostatic markers will require adjustment for confounding variables by stratified sub-analysis or multiple regression.

4) Results, Discussion and Conclusion

Invariably, a new statistical analysis will be required in the process of revising this manuscript. Hence, the entirety of the results, discussion and conclusion will require re-writing after all the already highlighted issues in concept, methods and analysis are addressed.

5) References, Tables & Figures

The numbered references can be better organised and presented, as some of the listed of references lacks appropriate content required by standard referencing styles.

Table-1 and Table-2 do need to be separated. Both tables simply show the characteristics of the participants. Authors need to clarify if Figure-2 included all participants or a subset. Figure-3 will be best placed side-by-side with the line graph for the non-diabetic group. All the figures should be better modified, improved and re-presented, as necessary.
