# Supplementary material for: Altered coagulation and platelet indices in Yemeni patients with type 2 diabetes mellitus: A conflict-affected population
Source: PLOS Glob Public Health. 2026 Jan 21;6(1):e0005173. doi: 10.1371/journal.pgph.0005173 (PMC12822953; doi:10.1371/journal.pgph.0005173)
Supplement: Attachment — Submitted filename: point to point response.pdf [file pgph.0005173.s006.pdf]

Response to Editors and Reviewers

Manuscript ID: PGPH-D-25-02423

Title:Altered Coagulation and Platelet Indices in Yemeni Patients with Type 2 Diabetes Mellitus: A Conflict-Affected Population

To: Paolo Ivo Cavoretto, MD PhD, Academic Editor, PLOS Global Public Health

CC:globalpubhealth@plos.org

Dear Dr. Cavoretto and Esteemed Reviewers,

We wish to extend our sincere gratitude for the opportunity to revise our manuscript and for the invaluable, constructive feedback provided by the reviewers and yourself. The comments were insightful and have been instrumental in guiding a comprehensive revision that has significantly strengthened the methodological rigor, clarity, and overall quality of our work. We have addressed all points raised in a point-by-point manner below and are confident that the revised manuscript now fully meets the high standards of PLOS Global Public Health.

Response to Journal Requirements

1. Ethics Statement: The Ethics Statement has been moved to the beginning of the Methods section under the subheading '2.1 Ethical Considerations' (Page 5, Lines 1-4).

2. Figure Files: All figure files have been converted to the required .tif format using the PLOS NAAS tool and now comply with the journal's resolution and formatting guidelines.

3. Supporting Information Legends: A complete list of legends for all Supporting Information files has been added after the References list (Page 21).

4. Data Availability: The minimum dataset required to replicate our findings has been deposited in the Open Science Framework (OSF) repository and is now publicly accessible without restriction at: https://osf.io/9f47q.

Response to Reviewer 1

We thank Reviewer 1 for their positive assessment and highly constructive suggestions, which have greatly enhanced the context and depth of our manuscript.

· **Comment 1.1 (Title): The phrase "The Paradox of War" in the title is intended to highlight the unique and complex interplay between the conflict environment and the observed hemostatic abnormalities... Suggestions for Improved Titles...**

· Response: We thank the reviewer for this insightful suggestion. We have changed the title to a more precise and less sensationalized version: "Altered Coagulation and Platelet Indices in Yemeni Patients with Type 2 Diabetes Mellitus: A Conflict-Affected Population." This new title accurately reflects the study's content while maintaining the relevant context.

· Comment 1.2 (Recent Literature): Include recent studies on coagulation abnormalities... please add the following recent study from the Middle East.

· Response: We have incorporated the suggested recent study from the Middle East (Al Ali et al., 2025) as reference [10]. Its findings regarding platelet indices and their prognostic value are now discussed in the Introduction (Page 4, Lines 8-11) and integrated into the regional comparison within the Discussion (Page 13, Lines 1-12).

· Comment 1.3 (Conflict Insights): Provide more insights into how conflict-related stress, malnutrition, and healthcare limitations specifically influence coagulation pathways beyond laboratory results.

· Response: We have enriched the Discussion to more cautiously explore these potential mechanisms (e.g., Page 11, Lines 19-25; Page 12, Lines 1-2). We now explicitly frame these as plausible hypotheses influenced by the context, rather than established conclusions, and directly link this to the expanded Limitations section.

· Comment 1.4 (Literature Support): Support your observation of prolonged PT and shortened APTT with previous literature reporting similar or contrasting patterns...

· Response: The Discussion now more thoroughly situates our findings within the global and regional literature. We explicitly compare our prolonged PT with reports from other resource-limited settings and contrast it with studies showing shortened PT, analyzing potential reasons for these discrepancies (Page 11, Lines 10-18).

· Comment 1.5 (Mechanisms): Elaborate on potential mechanisms behind the paradoxical coagulation profile, referencing studies on vitamin K deficiency, hepatic function, and inflammatory cytokines.

· Response: We have expanded the discussion of potential mechanisms, including vitamin K deficiency and the effects of metformin, while carefully noting that these are proposed explanations for which we lack direct measurements (Page 11, Lines 19-25). The concurrent shortening of APTT and its link to inflammatory cytokines and factors like FVIII/vWF is also discussed in greater detail (Page 12, Lines 3-10).

· Comment 1.6 (Limitations): Clearly acknowledge limitations like the cross-sectional design, lack of specific coagulation factor testing, and generalizability.

· Response: We have substantially expanded the Limitations section (Page 14, Line 14 to Page 15, Line 25) into a detailed, numbered list that explicitly addresses the cross-sectional design, unmeasured confounders (nutrition, specific vitamins, coagulation factors), medication data granularity, generalizability, and laboratory

constraints.

Response to Reviewer 2

We are profoundly grateful to Reviewer 2 for their exceptionally thorough and critical methodological review. Their comments challenged us to fundamentally re-evaluate and significantly strengthen our manuscript's scientific rigor. In direct response to the core concern, we have removed speculative narratives linking our findings directly to war-induced malnutrition. The revised manuscript is now firmly grounded in our empirical data and robust statistical analyses.

· **Comment 2.1 (Title & Abstract): The title should be re-written... The conclusive statement is more aspirational than anything that can be specifically deduced...**

· Response: We have comprehensively revised both the title and abstract.

· The title has been changed as described above.

· The abstract now consistently describes a "case-control" study.

· The term "significant majority" has been replaced with the descriptive "A striking 86%."

· The conclusion has been revised to be a direct and defensible inference from our findings, advocating for integrated screening rather than specific interventions we did not test.

· **Comment 2.2 (Introduction & Objectives): Revamp argument to be coherent with literature... remove conflict speculation... Streamline repetitive study aim statements.**

· Response: We have thoroughly revamped the Introduction.

· We corrected the narrative regarding PT in T2DM and removed unsupported claims.

· The speculative narrative about conflict effects has been replaced with a more measured description of the setting.

· The study objectives have been streamlined to be precise and focused on the parameters we actually measured.

· **Comment 2.3 (Methods - Major Revisions):**

· **Comment 2.3a (Study Design): The authors cannot be ambivalent about what study design was used...**

· Response: We unequivocally define the study as a "case-control" design throughout the manuscript (Page 5, Line 11). Terminology has been standardized

accordingly.

· **Comment 2.3b (Sampling): To claim simple random sampling was truly done... the authors were silent about the source of these "healthy controls".**

· Response: We have replaced "simple random sampling" with an accurate description. For cases, we used "consecutive sampling" from outpatient clinics (Page 6, Lines 8-12). For controls, we explicitly state they were recruited "from the same communities through health awareness campaigns" and detail the health-based inclusion criteria (Page 6, Lines 13-17).

· **Comment 2.3c (Contextual Challenges): "These contextual factors are... active determinants..." is a causal claim without measurement.**

· Response: We completely agree. The standalone "Contextual Challenges" section has been removed. This information has been appropriately integrated into the revised Limitations section (Page 15, Lines 8-12) as factors that may have influenced the research environment, without making causal claims about the results.

· **Comment 2.3d (Medication Data): ...use of metformin... was not measured.**

· Response: We now explicitly report and analyze metformin use. Data was collected showing 80% (112/140) of patients were on metformin therapy (Page 6, Lines 21-25), and this descriptive data is now included in the revised Table 1. The variable was subsequently included as a key covariate in our multivariable regression models (Page 9, Lines 20-25; Table 3).

· **Comment 2.3e (Statistical Analysis): The statistical analysis narrative is generic... will require adjustment for confounding variables.**

· Response: This is a cornerstone of our revision. We have completely overhauled the Statistical Analysis section (Page 8, Line 18 to Page 9, Line 25). We now detail the use of:

· Multiple Linear Regression with three specified models (unadjusted, age/sex-adjusted, fully adjusted).

· Effect sizes (Cohen's d) for all group comparisons.

· A clear rationale for each statistical test used.

- **Comment 2.3f (Sample Size Calculation): The reviewer noted the need for a specific statistical reference for the sample size calculation.**

- Response: We have updated the 'Sample Size Justification' paragraph in the Methods section (Page 7, Lines 3-7) to specify that the calculation was performed using GPower software (version 3.1.9.7), providing the requested methodological detail and transparency.

· **Comment 2.4 (Results, Discussion, Conclusion): "Invariably, a new statistical**

analysis will be required... the entirety of the results, discussion and conclusion will require re-writing."

· Response: We have undertaken this comprehensive rewrite.

· Results: The Results section now includes the new multivariable regression analysis (Table 3), reports effect sizes, and presents all findings with greater statistical precision (Pages 10-13).

· Discussion: The Discussion has been fundamentally restructured to focus on our empirical findings and their clinical implications. We discuss the robustness of the PT/APTT findings after adjustment for confounders, the strong correlation between HbA1c and MPV, and provide a balanced comparison with regional studies. Speculative explanations have been replaced with cautious hypotheses explicitly linked to the study's limitations.

· Conclusion: The Conclusion is now a direct and concise summary of our findings and their practical implications for clinical monitoring, devoid of overstatement.

· Comment 2.5 (References, Tables & Figures): The numbered references can be better organised... Tables 1 and 2 do need to be separated.

· Response:

· All references have been verified and formatted to a consistent standard.

· Tables 1 and 2 are now presented as separate tables.

· A new Table 3 for the regression analysis has been added.

· All figures have been refined with improved legends and are submitted in the correct format.

Summary of Major Revisions

1. Methodological Clarification and Rigor: The study is now explicitly and consistently presented as a case-control study. Sampling methods are accurately described, and a comprehensive multivariable regression analysis has been added to control for key confounders.

2. Shift from Speculation to Evidence-Based Interpretation: The manuscript no longer speculatively attributes findings to war or malnutrition. The discussion is firmly grounded in the data, with unmeasured factors appropriately discussed as limitations.

3. Enhanced Statistical Transparency: The analysis now includes effect sizes, confidence intervals, and a robust adjustment for covariates, greatly strengthening the validity of our conclusions.

4. Comprehensive and Transparent Limitations: A greatly expanded limitations section provides an honest and detailed account of the study's constraints, including unmeasured confounders and design limitations.

5. Formal Compliance: All journal requirements regarding ethics statements, figure formats, data availability, and supporting information have been met.

We believe the reviewers' critical engagement has transformed our manuscript into a stronger, more reliable contribution to the literature. We are deeply appreciative of their time and expertise.

Thank you for considering our revised manuscript for publication in PLOS Global Public Health.

Sincerely,

Naif Taleb Ali (Corresponding Author)

On behalf of all co-authors

Email: n.taleb@ust.edu

ORCID:0000-0002-2266-1569
