# Supplementary material for: Altered coagulation and platelet indices in Yemeni patients with type 2 diabetes mellitus: A conflict-affected population
Source: PLOS Glob Public Health. 2026 Jan 21;6(1):e0005173. doi: 10.1371/journal.pgph.0005173 (PMC12822953; doi:10.1371/journal.pgph.0005173)
Supplement: Attachment — Submitted filename: PGPH-D-25-02423R1_review Report.pdf [file pgph.0005173.s007.pdf]

Review of the Revised Version (PGPH-D-25-02423R1)

Primarily, I wish to commend the author's prompt submission of their revised manuscript, with substantial changes made but, the following issues on the draft document will require some more attention before acceptance for publication:

1) *Title, Abstract & Introduction*

Generally, the authors should be reminded that they are writing a scientific paper, and not some opinion piece. In other words, their textual narrative should be evidently supported by the numbers. More importantly, the statistical procedures generating the numbers should be correctly done to make the numbers credible to the potential users of their hard-earned research output. ***For example, the 0.52 correlation coefficient reported under result in the abstract is NOT considered "strong positive correlation" by any classification system. That is moderate correlation, at best***. So, it will be more helpful to their hard work to honestly interpret their methods and findings, instead of the needless exaggerations, and sensational adjectives. This is a major concern that apply to every aspect of the methods, results and discussion sections of this manuscript.

2) *Methods, Results and Discussion*

The authors have corrected their study design as a case control study, with useful adjustment. As previously advised, their reporting attempt will be more optimized if they consult the STROBE guidelines and follow its recommendations.

Strikingly, the authors still did not define their main outcome(s) of interest, primary predictor(s), such that we can consider all other variables as covariates. Doing this will enhance their attempt at using appropriate statistical techniques and interpreting results more correctly, especially the multiple regression modelling. For example, the study objective is ambivalent about whether glycaemic level is the outcome or it is coagulation or platelet parameters. This should be clarified in the definition of variables in the methods section so that it can be clear what specific outcome(s) is being modelled, the main predictor(s) that we are estimating its effect on the outcome, such that other covariates are adjusted for their confounding, mediating or modifying effects.

If coagulation and platelet parameters are the outcomes of interest by the consistent narrative in the manuscript text, why are the authors reporting beta-coefficients and p-values for their effects, as shown in table 3? What outcome was this coagulation parameters having effects on? It will be counter-intuitive if the authors were trying to predict glycaemic levels with coagulation parameters in their model. Why? How? What is the logic?

Also, there are only eight measured variables reported in this study, as shown in tables 1 and table 2. These are few enough for the authors to be less generic about their statistical technique for handling each variable. ***After the previous review recommendations, the authors claimed to have adjusted their multiple regression model for age, sex, metformin use, hypertension, and cardiovascular history. The age and sex distribution of the participants are shown in table 1 but, I could not find the numbers for the other three variables.*** If they measured and

adjusted for these variables, it is more credible to show their descriptive numbers, even if they wish to leave these covariates out of the multiple regression model table 3.

“Multivariable regression” is a broad theme that is often used to describe a large group of simple and multiple regression procedures that apply to different ***“univariate or multivariate OUTCOME variable types”*** with strictly different dataset terms and conditions. I hope the authors can stick to using the specific term “multiple linear regression” that was conducted in this study in their narrative, rather than “multivariable regression”. Also, the authors made three models for unclear reasons. I could decipher the adjustment made in model 2 and model 3, as stated. What does the “unadjusted model 1” contain? There should be at least one outcome and predictor in it. State them. Lastly, I will advise the optional reporting of the effect sizes for the covariates included in the multiple regression model, as they are of interpretative value, as well as the main predictors.

Finally, the enormous work put into this research will be most rewarding to all stakeholders if the authors will consider meticulous attention to their methodology, statistical procedures and correct interpretation. We all know that findings and conclusions of research are ONLY as valid or credible as the methods and statistical techniques applied. The authors should take another look at my previous review recommendations for other pending concerns.
