# Supplementary material for: Altered coagulation and platelet indices in Yemeni patients with type 2 diabetes mellitus: A conflict-affected population
Source: PLOS Glob Public Health. 2026 Jan 21;6(1):e0005173. doi: 10.1371/journal.pgph.0005173 (PMC12822953; doi:10.1371/journal.pgph.0005173)
Supplement: Attachment — Submitted filename: Respone to Reviewers.pdf [file pgph.0005173.s008.pdf]

RESPONSE TO REVIEWERS

Manuscript ID: PGPH-D-25-02423R1

Title: **Altered Coagulation and Platelet Indices in Yemeni Patients with Type 2 Diabetes Mellitus: A Conflict-Affected Population**

We thank the Academic Editor and Reviewers for their thoughtful comments and constructive feedback on our manuscript. We have carefully addressed all points raised and believe the revisions have significantly strengthened our manuscript. Below we provide a point-by-point response to all comments.

RESPONSES TO REVIEWER COMMENTS

Reviewer 1:

Comment:"Thank you for addressing all of my suggestions, there is minor correction in references no 10, the author name are not belong to the references, see the correct citation as follow: Obaid Al Ali, Maryam Ahmed, Khalid Abdelsamea Mohamedahmed, and Asaad Ma Babker. 'Role of platelet indices and vitamin D in forecasting deterioration of glycemic control and vascular complications in type 2 diabetes.' Italian Journal of Medicine 19, no. 1 (2025)."

Response: We thank Reviewer 1 for their positive feedback and for identifying this citation error. The reference has been corrected as requested.

Changes made:

· Reference 10 has been updated with the correct authorship and citation details in the reference list.

Reviewer 2:

We sincerely thank Reviewer2 for their thorough assessment and valuable suggestions that have significantly improved the methodological rigor and statistical reporting of our manuscript.

Comment 1: "Generally, the authors should be reminded that they are writing a scientific paper, and not some opinion piece. In other words, their textual narrative should be evidently supported by the numbers. More importantly, the statistical procedures generating the numbers should be correctly done to make the numbers credible to the potential users of their hard-earned research output. For example, the 0.52 correlation coefficient reported under result in the abstract is NOT considered 'strong positive correlation' by any classification system. That is moderate

correlation, at best. So, it will be more helpful to their hard work to honestly interpret their methods and findings, instead of the needless exaggerations, and sensational adjectives. This is a major concern that apply to every aspect of the methods, results and discussion sections of this manuscript."

Response: We thank the reviewer for this important correction regarding statistical interpretation. We have revised our description of correlation coefficients throughout the manuscript to ensure accurate and conservative interpretation.

Changes made:

· Abstract: "strong positive correlation" changed to "moderate positive correlation"

· Results section 3.5: Updated correlation description from "strong" to "moderate"

· Discussion section 4.2: Corrected correlation interpretation

· Comprehensive review of all sections to eliminate exaggerated language and ensure precise, evidence-based interpretations

Comment 2: "The authors have corrected their study design as a case control study, with useful adjustment. As previously advised, their reporting attempt will be more optimized if they consult the STROBE guidelines and follow its recommendations."

Response: We thank the reviewer for this suggestion regarding reporting standards.

Changes made:

· Methods section 2.2: Added statement confirming adherence to STROBE guidelines

· Supporting Information: Included completed STROBE checklist as S1 File

Comment 3: "Strikingly, the authors still did not define their main outcome(s) of interest, primary predictor(s), such that we can consider all other variables as covariates. Doing this will enhance their attempt at using appropriate statistical techniques and interpreting results more correctly, especially the multiple regression modelling. For example, the study objective is ambivalent about whether glycaemic level is the outcome or it is coagulation or platelet parameters. This should be clarified in the definition of variables in the methods section so that it can be clear what specific outcome(s) is being modelled, the main predictor(s) that we are estimating its effect on the outcome, such that other covariates are adjusted for their confounding, mediating or modifying effects."

Response: We appreciate this critical insight regarding variable definition and statistical modeling.

Changes made:

· Methods section 2.3: Added "Variable Definition" paragraph specifying:

· Primary outcomes: coagulation parameters (PT, APTT) and platelet indices (MPV, PDW)

· Main predictor: T2DM status (case/control)

· Covariates: age, sex, metformin use, hypertension, cardiovascular history

· Statistical Analysis section 2.6: Clarified regression model specifications and relationships between variables

Comment 4: "If coagulation and platelet parameters are the outcomes of interest by the consistent narrative in the manuscript text, why are the authors reporting beta-coefficients and p-values for their effects, as shown in table 3? What outcome was this coagulation parameters having effects on? It will be counter-intuitive if the authors were trying to predict glycaemic levels with coagulation parameters in their model. Why? How? What is the logic?"

Response: This is an excellent point that highlights the need for clarity in our statistical reporting.

Changes made:

· Results section 3.4: Added explicit statement: "The outcomes for these models were PT and APTT as continuous dependent variables. T2DM status was the primary predictor, with other variables treated as covariates."

· Table 4: Updated headings and structure to clearly reflect the relationship between predictors and outcomes

· Statistical Analysis section 2.6: Clarified the directionality and purpose of all regression models

Comment 5: "Also, there are only eight measured variables reported in this study, as shown in tables 1 and table 2. These are few enough for the authors to be less generic about their statistical technique for handling each variable. After the previous review recommendations, the authors claimed to have adjusted their multiple regression model for age, sex, metformin use, hypertension, and cardiovascular history. The age and sex distribution of the participants are shown in table 1 but, I could not find the numbers for the other three variables. If they measured and adjusted for these variables, it is more credible to show their descriptive numbers, even if they wish to leave these covariates out of the multiple regression model table 3."

Response: We thank the reviewer for identifying this omission in our descriptive statistics.

Changes made:

· New Table 2: Added "Clinical Characteristics and Comorbidities of Study Participants" showing distributions of all covariates used in adjusted models (hypertension, cardiovascular history, metformin use, smoking, exercise)

· Results section 3.1: Updated text to reference this new table and describe the distribution of all covariates

Comment 6: "'Multivariable regression' is a broad theme that is often used to describe a large group of simple and multiple regression procedures that apply to different 'univariate or multivariate OUTCOME variable types' with strictly different dataset terms and conditions. I hope the authors can stick to using the specific term 'multiple linear regression' that was conducted in this study in their narrative, rather than 'multivariable regression'. Also, the authors made three models for unclear reasons. I could decipher the adjustment made in model 2 and model 3, as stated. What does the 'unadjusted model 1' contain? There should be at least one outcome and predictor in it. State them. Lastly, I will advise the optional reporting of the effect sizes for the covariates included in the multiple regression model, as they are of interpretative value, as well as the main predictors."

Response: We appreciate this detailed statistical guidance regarding terminology, model specification, and complete results reporting.

Changes made:

· Terminology: Consistently replaced "multivariable regression" with "multiple linear regression" throughout manuscript

· Model Specification: Clearly defined all three models in Methods section 2.6:

· Model 1 (Unadjusted): T2DM status as sole predictor of coagulation parameters

· Model 2: Adjusted for age and sex

· Model 3 (Fully adjusted): Additional adjustment for metformin use, hypertension, and cardiovascular history

· Complete Results Reporting: Updated Table 4 now includes:

· Beta coefficients with 95% CIs for T2DM status across all models

· Beta coefficients with 95% CIs for all covariates in adjusted models

· Comprehensive reporting of all variables as recommended

Comment 7: "Finally, the enormous work put into this research will be most rewarding to all stakeholders if the authors will consider meticulous attention to their methodology, statistical procedures and correct interpretation. We all know that findings and conclusions of research are ONLY as valid or credible as the methods and statistical techniques applied. The authors should take another look at my previous review recommendations for other pending concerns."

Response: We sincerely thank the reviewer for emphasizing the importance of methodological rigor. We have thoroughly reviewed all previous recommendations and implemented comprehensive revisions to enhance the validity and credibility of our findings.

Changes made:

· Comprehensive review of all statistical procedures and interpretations

· Enhanced methodological descriptions throughout

· Added STROBE checklist to ensure complete reporting

· Verified all statistical analyses and corrected interpretations as needed

ADDITIONAL CHANGES IMPLEMENTED:

1. Data Availability Statement: Enhanced to confirm all variables are publicly accessible

2. Table and Figure Renumbering: Updated all references following addition of new tables

3. Comprehensive Language Review: All sections reviewed to ensure accurate interpretation and eliminate exaggerated language

4. Reference Correction: Updated reference 10 as per Reviewer 1's suggestion

CONCLUSION

We believe these revisions have substantially improved our manuscript and addressed all concerns raised by the reviewers. The methodological clarity, statistical reporting, and interpretation of findings have been significantly enhanced. We thank the editor and reviewers for their time and expertise in evaluating our work.

Sincerely,

Naif Taleb Ali

On behalf of all co-authors
