# Supplementary material for: Altered coagulation and platelet indices in Yemeni patients with type 2 diabetes mellitus: A conflict-affected population
Source: PLOS Glob Public Health. 2026 Jan 21;6(1):e0005173. doi: 10.1371/journal.pgph.0005173 (PMC12822953; doi:10.1371/journal.pgph.0005173)
Supplement: Attachment — Submitted filename: Respone_to_Reviewers_auresp_3.pdf [file pgph.0005173.s009.pdf]

Response to Reviewers

Dear Dr. Cavoretto and Reviewers,

Thank you for your valuable feedback on our manuscript, Altered Coagulation and Platelet Indices in Yemeni Patients with Type 2 Diabetes Mellitus: A Conflict-Affected Population. We have carefully addressed all the points raised and have made the necessary revisions to the manuscript. Our point-by-point responses are detailed below. All changes have been highlighted in the submitted Revised Manuscript with Track Changes. A clean version is also provided.

Response to Additional Editor Comments:

1. Comment:

Abstract: A strong positive correlation was observed between HbA1c and MPV ($r = 0.52$, $p < 0.001$). This is moderate correlation at best, please revise in the abstract as well as in the main text and conclusions.

Our Response:

We have revised all instances where the correlation between HbA1c and MPV was described as strong. In the Abstract, Results (section 3.5), and Discussion (sections 4.3 ,4.2, and 4.4), the term “strong” has been consistently changed to moderate.

2. Comment:

Please smooth the conclusion as association does not imply causation.

Our Response:

We have revised the Conclusion to clarify that the findings demonstrate association, not causation. The phrasing now emphasizes observed associations and highlighted relationships to avoid any implication of causality (section 5).

3. Comment:

There are inconsistencies in the manuscript: the chapter Sample Size Justification is written twice (page 5) please revise.

Our Response:

The duplicate Sample Size Justification subsection on page 5 has been removed.

The remaining single Sample Size Justification paragraph (under 2.3 Study Participants and Sampling) now clearly includes the calculation rationale and reference to G*Power software without redundancy.

4. Figure Format Compliance

As recommended, we have used PLOS's NAAS tool to process our figure files. The figures (1–3) have been successfully converted to TIFF format and meet all specified requirements for resolution and print size. The processed files are included with this submission.

All requested revisions have been implemented. We believe the manuscript has been strengthened and now fully meets the journal's criteria. Thank you for the opportunity to improve our work.

Sincerely,

Naif Taleb Ali, on behalf of all authors
